# Post-transplant lymphoproliferative disease after pediatric kidney transplant

**DOI:** 10.3389/fped.2022.1087864

**Published:** 2022-12-07

**Authors:** Rosanna Fulchiero, Sandra Amaral

**Affiliations:** ^1^ Department of Nephrology, Children's Hospital of Philadelphia, Philadelphia, PA, United States; ^2^ Department of Biostatistics, Epidemiology and Informatics, University of Pennsylvania, Philadelphia, PA, United States

**Keywords:** pediatrics, kidney transplant, Epstein-Barr virus infection, post-transplant lymphoproliferative disease, malignancy

## Abstract

Post-transplant lymphoproliferative disease (PTLD) is the most common malignancy complicating solid organ transplantation (SOT) in adults and children. PTLD encompasses a spectrum of histopathologic features and organ involvement, ranging from benign lymphoproliferation and infectious-mononucleosis like presentation to invasive neoplastic processes such as classical Hodgkin lymphoma. The predominant risk factors for PTLD are Epstein-Barr virus (EBV) serostatus at the time of transplant and the intensity of immunosuppression following transplantation; with EBV-negative recipients of EBV-positive donor organs at the highest risk. In children, PTLD commonly presents in the first two years after transplant, with 80% of cases in the first year, and over 90% of cases associated with EBV-positive B-cell proliferation. Though pediatric kidney transplant recipients are at lower risk (1–3%) for PTLD compared to their other SOT counterparts, there is still a significant risk of morbidity, allograft failure, and an estimated 5-year mortality rate of up to 50%. In spite of this, there is no consensus for monitoring of at-risk patients or optimal management strategies for pediatric patients with PTLD. Here we review pathogenesis and risk factors for the development of PTLD, with current practices for prevention, diagnosis, and management of PTLD in pediatric kidney transplant recipients. We also highlight emerging concepts, current research gaps and potential future developments to improve clinical outcomes and longevity in these patients.

## Introduction

Post-transplant lymphoproliferative disease (PTLD) is the most common malignancy complicating solid organ transplantation (SOT) in adults and children (1). Incidence is variable across allograft type, with a relatively low incidence of 1%–3% in pediatric kidney transplant recipients compared with other SOT patients: intestinal and multi-organ (5%–20%), heart and lung (2%–10%) and liver (1%–5%) (2–5). Generally, PTLD in SOT patients has a bimodal presentation, with “early” PTLD developing within the first two years of transplant, and “late” PTLD developing 5–10 years after transplant (6, 7). The majority of PTLD is “early” and related to Epstein-Barr virus (EBV) infection; with over 80% of pediatric cases presenting in the first year after transplant (8, 9) during the most intensive period of T-cell immunosuppression.

While reduction of immunosuppression is a cornerstone of therapy, patients often require additional treatments, including rituximab, chemotherapy, and adoptive immunotherapy, with radiation and surgical therapy reserved for select cases. Current literature in pediatric PTLD is limited to case series and small studies and many practice patterns for treatment are adopted from the adult literature, as outlined in this review. Importantly, PTLD is associated with significant morbidity and mortality, and at present, there is no consensus for monitoring of at-risk patients or optimal management strategies for pediatric patients with PTLD. Early recognition of risk factors with close attention to prevention efforts and prompt intervention is paramount for improving survival.

Here we review pathogenesis and risk factors for the development of PTLD, with current practices for prevention, diagnosis, and management of PTLD in pediatric kidney transplant recipients. We also highlight emerging concepts, current research gaps and potential future developments to improve clinical outcomes and longevity in these patients.

## Pathogenesis and risk factors

Though there are several reported risk factors for the development of PTLD, the two major risk factors in pediatric kidney transplant recipients are: EBV-serostatus at the time of transplant and the intensity of T-cell immunosuppression following transplantation (10).

Over 90% of pediatric PTLD is due to EBV positive B-cell proliferation in the setting of immunosuppression and decreased T-cell immune surveillance. Typically, acute EBV infection results in a polyclonal expansion of B-cells containing the virus. In healthy, immunocompetent hosts, EBV infected B-cells are rapidly eliminated by EBV-specific cytotoxic T-cells. However, in the setting of high-dose T-cell immunosuppression (such as thymoglobulin), cytotoxic T-cell function is diminished, resulting in uncontrolled proliferation of EBV-infected B-cells and the development of PTLD (11, 12).

Patients who are at highest risk for PTLD are EBV-negative recipients of EBV-positive donor organs (13, 14). EBV infection is more common in adults than children, with approximately 90%–95% of adults showing serologic evidence of infection and acquired immunity at the time of transplantation. Conversely, children are more likely to be EBV-negative, and may acquire EBV from the donor organ and/or primary EBV infection post-transplant, putting them at increased risk for PTLD. This was highlighted in one study of 276 pediatric kidney transplant recipients in which EBV-negative recipients of EBV-positive donor organs demonstrated a 6-fold higher risk of PTLD (15). The highest incidence of PTLD occurs at primary EBV seroconversion due to *de novo* infection or when it is acquired from passenger lymphocytes in the graft (16). Both primary EBV infections in EBV-negative patients and reactivation in EBV-positive individuals with latent infection can lead to uninhibited growth of EBV-infected B-cells and the development of PTLD (17).

While there is a strong association between EBV and PTLD, not all patients with high EBV viral load will go on to develop PTLD. Furthermore, an estimated 20%–30% of PTLD cases are not associated with EBV (17, 18). The pathogenesis of EBV-negative PTLD is not well understood, but these cases typically present later, 7–10 years after transplantation, compared with the earlier presentation of EBV-positive PTLD (7, 19, 20). These inconsistencies complicate post-transplant monitoring protocols and pose challenges in managing patients with EBV-viremia. Regardless, prior studies in pediatric kidney transplant recipients have demonstrated up to 35% mortality from PTLD (21–23), highlighting the importance of monitoring, prompt diagnosis and intervention, as well as prevention.

## Prevention

As previously discussed, the predominant risk factors for development of PTLD are the degree of T-cell immunosuppression and development of EBV infection. Thus, prevention of PTLD is centered around minimizing risk by limiting immunosuppressive exposure and optimizing opportunities for early intervention with screening for EBV viremia.

### Reduction of immunosuppression

The role of immunosuppression after transplant is to prevent acute rejection and loss of the allograft while balancing the risk of treatment-related toxicities and complications such as infection and malignancy. High-level induction immunosuppression is required in the immediate post-transplant period to dampen the immune response to the allograft and prevent early rejection. Over time, as the risk of acute rejection decreases, chronic immunosuppression is lessened accordingly in an effort to promote long-term graft survival and reduce long-term toxicities. Conventional maintenance regimens generally consist of a combination of three immunosuppressive agents with different mechanisms of action based on widely adopted clinical practice guidelines (24). Though these regimens differ by patient, transplant center and geographic region, most pediatric patients will receive a calcineurin inhibitor (tacrolimus or cyclosporine), an antimetabolite (mycophenolate mofetil, azathioprine) and varying degrees of prednisone based on immunological risk of rejection and center-specific protocols.

It is not clear whether any particular immunosuppressive regimen is more protective for PTLD than another. Some studies have suggested protective effects of mTOR inhibitors due to their anti-proliferative effects, but other studies suggest potential increased risk of PTLD with mTOR introduction (25–28). Historically, increased risk of PTLD has been observed in patients with higher target tacrolimus trough concentrations (10, 14, 29, 30). In general, current practice patterns have focused on minimization of overall immunosuppression which is favorable for reducing the risk of PTLD.

Steroid reduction or withdrawal is another key area of interest (31). Though several small randomized controlled trials have evaluated steroid avoidance or withdrawal protocols in pediatric kidney transplant patients, most have had insufficient sample sizes with conflicting results. A recent meta-analysis shows justification for steroid avoidance/withdrawal in select pediatric kidney transplant recipients because of benefits in post-transplant growth with minimal effects on risk of acute rejection, and graft function (32). Though it was not a primary focus of the analysis, there was no difference in PTLD cases between groups. This suggests a potential future benefit to reduction of corticosteroids in select patient populations at increased risk for PTLD, though more research in larger patient populations is needed.

### EBV monitoring and preemptive treatment of EBV-reactivation

Monitoring of the EBV viral load is essential for early detection of current or impending PTLD in pediatric kidney transplant patients, and higher viral loads are associated with increased predilection for disease (33–38). In line with current society guidelines, many centers monitor high risk kidney transplant recipients (donor positive/recipient negative) for EBV viremia at varying intervals following transplant, starting once in the first week after transplant, then monthly for the first 3–6 months, and every 3 months until the end of the first year; with additional testing as necessary, particularly after anti-rejection therapy (24). Studies have shown that frequent monitoring of EBV per the aforementioned protocol, with more frequent testing (every 2 weeks) once EBV is >1,000 genome equivalents/mL, may lower the incidence of PTLD, especially in EBV seronegative patients (39, 40). Frequent monitoring allows for an earlier opportunity for intervention and preemptive treatment with increased likelihood for favorable outcomes.

### Other considerations: antiviral prophylaxis and vaccination

Data regarding the use of antiviral prophylaxis for the prevention of PTLD is limited with conflicting results. In 2005, a retrospective multicenter case-control study of pediatric and adult kidney transplant recipients demonstrated a decreased risk of PTLD by 38% for every 30 days of treatment with ganciclovir in the first 12 months after transplant (41). This effect was not sustained in a larger, more recent systematic review which showed no significant difference in the rate of EBV-associated PTLD in high-risk, EBV naïve SOT recipients who received prophylaxis compared with those who didn't, regardless of age or type of organ transplant (42). Irrespective of these results, most kidney transplant recipients are exposed to antiviral prophylaxis against cytomegalovirus for at least the first three months after transplant, and it is possible this antiviral exposure is playing an unrecognized role in the prevention of early-onset PTLD.

Research on EBV vaccine development has been longstanding, however progress has been hampered by the complex nature of the EBV replication cycle and vast range of host cells. In May 2022, the National Institutes of Health in the United States announced a phase I clinical trial to evaluate the safety and immunogenicity of an investigational EBV gp350-Ferritin nanoparticle vaccine with a saponin-based Matrix-M adjuvant in healthy adults (43, 44). Results will be eagerly anticipated and have potential to significantly reduce the incidence of PTLD for transplant recipients.

## Diagnosis

The diagnosis of PTLD involves a multifaceted approach with histologic confirmation in the context of high clinical suspicion.

Clinically, signs and symptoms of PTLD are highly variable, ranging from asymptomatic to life threatening disease, and depend in part on the specific category of PTLD and the organ(s) involved. Patients will typically have non-specific constitutional symptoms such as fever, fatigue and weight loss (7, 45). Frequently, PTLD presents with extra-nodal masses in various organ systems: abdominal involvement in 60%–70%; thorax involvement in 45%–65%, head and neck in 20%–30% and central nervous system disease in up to 20%–25% (46, 47). Additionally, 20%–25% may have infiltrative lesions in the allograft and present with allograft dysfunction. Thus, additional symptoms may reflect primary or secondary organ dysfunction from mass compression of surrounding structures.

Patients may also present with laboratory abnormalities similar to non-transplant patients with lymphoproliferative disorders, such as: anemia, thrombocytopenia, leukopenia, elevated serum lactate dehydrogenase, hypercalcemia or hyperuricemia (48). There may also be radiologic evidence of a mass or positive positron emission tomography (PET) scanning indicating possible metabolically active areas, which also favors the diagnosis.

Although an elevated EBV viral load should raise suspicion for EBV-positive PTLD, this is not sufficient for diagnosis. In patients with a high clinical suspicion for PTLD (based on symptoms, EBV viremia, or both), diagnosis and classification require tissue biopsy, with excisional biopsy of a suspicious lesion when possible (49). And, while the absence of EBV in the peripheral blood makes PTLD less likely, it does not completely exclude the diagnosis (50).

### Pathology and classification

The most recent World Health Organization guidelines divide PTLD into four main categories based on morphologic, immunophenotypic, genetic and clinical features (51). Identifying the category in which a patient belongs is paramount for guiding management as outlined in Table 1. Here we review a brief description of the four histopathologic categories.

**Table 1:** Overview of management strategies based on classification of PTLD.

PTLD Category	General management strategies
Early lesions/non-destructive PTLD • Plasmacytic hyperplasia • Infectious mononucleosis-like • Florid follicular hyperplasia	Reduction of IS
Polymorphic	Reduction of IS + rituximab (if CD20+) *Chemotherapy/surgery in certain patients
Monomorphic • Diffuse large B cell • Burkitt lymphoma • Plasma cell neoplasm • Peripheral T-cell lymphoma, NOS • EBV + T/NK-cell lymphoma	CD20 (±) PTLD: Reduction of IS + rituximab ± chemotherapy (CHOP) CD20 (−) PTLD and/or not candidate for rituximab: Reduction of IS + combination chemotherapy (CHOP) *Surgery is reserved for patients with perforation or obstruction *EBV-CTLs may be preferred over chemotherapy in some cases
Classic Hodgkin lymphoma-like	Chemotherapy ± radiation per protocols for classic HL

The first category of PTLD is non-destructive and encompasses three patterns: plasmacytic hyperplasia, infectious mononucleosis-like and florid follicular hyperplasia. These are considered early lesions with benign proliferations.

The remaining three categories are considered neoplastic processes and the presence of a lymphoid tumor in combination with two of the three following features confirm a diagnosis of PTLD: disruption of underlying tissue architecture by a lymphoid proliferation, presence of mono- or oligoclonal lymphoid cell populations, and EBV infection of many cells.

Monomorphic PTLD meets the criteria for a non-Hodgkin B cell or T/NK cell lymphoma with monoclonal malignant cells. The majority are B cell lymphomas (most commonly diffuse large B cell lymphoma) but can include Burkitt lymphoma, plasma cell neoplasm, peripheral T-cell lymphoma not otherwise specified or EBV + T/NK cell lymphoma.

Polymorphic PTLD has a pleomorphic lymphoid infiltrate that does not fill criteria for one of the aforementioned B cell or T/NK cell lymphomas above.

Classical Hodgkin lymphoma-like PTLD fulfills the criteria required for the diagnosis of classic HL and is the least common form of PTLD.

## Management

The overarching goals for PTLD management are eradication of the PTLD and preservation of allograft function. These priorities often involve conflicting treatment approaches, and typically one goal will take precedence over the other based on the specific needs of the patient. For example, a mainstay of PTLD eradication involves reduction of immunosuppression, but this increases the risk of graft rejection and failure.

The main approaches to PTLD management largely depend on the PTLD subtype but nuances may vary from institution to institution. Here we review the most common PTLD treatment strategies, their respective indications, and the risks and benefits to each approach.

### Reduction of immunosuppression

Reduction of immunosuppression (RIS) is the cornerstone of management for all types of PTLD, with the goal of restoring EBV-specific cellular immunity without increasing the risk of acute rejection. This remains the most common practice at diagnosis of PTLD in pediatric SOT recipients (16, 24). Generally, immunosuppression is reduced to the lowest tolerable level, and at times can reach as low as 25%–50% of baseline therapy. Although there are no specific protocols for a graded decrease in IS, many centers reduce calcineurin inhibitors by 50% given prior published literature regarding the interplay between high serum tacrolimus troughs, EBV-viremia and development of PTLD (14, 29, 30). Antimetabolite therapy is often decreased in parallel or after initial reduction in CNI, with variable reduction or withdrawal of corticosteroids per center preference (7). As mentioned earlier, there is no specific immunosuppressive agent that is shown to be more or less PTLD-inducing, rather the overall degree of immunosuppression is the major risk factor.

Retrospective studies regarding the efficacy of RIS in adults and children are challenging to interpret due to study design and small sample sizes with conflicting results. Some studies suggest resolution of early PTLD lesions with RIS alone, but other studies suggest that many patients treated initially with RIS go on to require additional therapy, and up to 50% may have evidence of organ rejection during their reduced immunosuppression phase (52). Prospective study data in children is scarce, but adult solid organ transplant studies demonstrate a complete response rate of 37% from RIS alone; though this was balanced against a 32% rate of acute rejection in the same study population (53). RIS alone may be insufficient for treatment of PTLD, but early RIS in the setting of increasing EBV-viral load may prevent progression to PTLD and thus it remains first-line management in patients at risk for- and with confirmed PTLD (23, 40, 45).

### Immunotherapy with rituximab

Rituximab is an anti-CD20 monoclonal antibody and a reasonable therapeutic option for patients with CD20+ PTLD with residual disease despite RIS or for those who are not candidates for RIS.

Rituximab can be used alone or in conjunction with chemotherapy depending on the clinical circumstance. There are no current consensus guidelines for whom would benefit from both therapies and whether to administer them concurrently or sequentially. Patients with minimal symptoms and/or those who are not candidates for initial chemotherapy can begin with rituximab alone at a typical dosing regimen of 375 mg/m2 weekly for 3–4 doses. Adult SOT studies demonstrate response rates of 44%–79% with rituximab monotherapy and complete remission rates in 20%–55%, obviating the need for chemotherapy in many patients (7). There is a paucity of data for outcomes of rituximab monotherapy in pediatric patients with PTLD. One small study suggested complete remission rates of 70%–75% in pediatric SOT patients (54), and one study limited to pediatric kidney recipients demonstrated stable graft function and favorable graft survival with rituximab alone or in combination with chemotherapy (55); but more research with larger, prospective studies is necessary. Of note, rituximab imposes a risk of infusion reactions (fevers, rigors, hypotension), hepatitis B reactivation in patients with positive hepatitis B surface antigen or hepatitis B core antigen antibodies, and isolated neutropenia.

### Chemotherapy

Patients with CD20 + PTLD may receive chemotherapy in conjunction with rituximab in a regimen known as R-CHOP (rituximab plus cyclophosphamide, doxorubicin, vincristine and prednisone). The ideal time course of these therapies remains unknown; however, many authors suggest the addition of chemotherapy in patients who don't achieve an adequate response or complete remission after initial treatment with rituximab (56–58). These studies demonstrate improved response and remission rates when CHOP therapy was added to rituximab, with increased complete remission rates to 50%–65%. One phase II trial in pediatric patients with EBV + CD20+ PTLD showed a complete remission rate of 69% when receiving rituximab in combination with low-dose cyclophosphamide and prednisone, with 2-year event free survival (alive with functioning allograft and no PTLD) of 83% (59).

Patients with CD20- PTLD or those who are not candidates for rituximab may receive CHOP chemotherapy alone; and patients with classic Hodgkin lymphoma (HL) like PTLD will receive chemotherapy in accordance with HL protocols. There are no randomized trials comparing chemotherapy regimens in PTLD and some patients may require other regimens at the discretion of their physician or based on their personal risk of side effects. Indication for and selection of additional agents is beyond the scope of this review.

### Radiation or surgical therapy

Local therapy by surgery or radiation is limited to rare situations, such as treatment of local disease, symptomatic control, or palliative care. For disseminated or central nervous system disease, radiation therapy may be indicated in conjunction with chemotherapy (60, 61).

### Adoptive immunotherapy: EBV-specific cytotoxic T-cells

Adoptive cell therapy with EBV-cytotoxic T-lymphocytes (EBV-CTLs) involves transferring naturally occurring EBV-specific CTLs that can kill EBV-transformed B cells into recipients with EBV + PTLD (62). Up to this point, the majority of published research regarding use of EBV-CTLs has been in hematopoietic stem cell transplant patients (HSCT). Use of EBV-CTLs in solid organ transplant recipients is gaining popularity in the last decade, particularly in patients with treatment-refractory PTLD.

One systematic review of 36 adult patients demonstrated a 66% response rate, with rare adverse effects limited to mild, nonspecific symptoms (nausea, vomiting, fever, tachycardia) (63). Use of EBV-CTLs in pediatric SOT patients is limited, but published results mimic those of adult studies, with 80% remission rate and survival rates of 89% and 86% at 2 and 5 years respectively (64).

Previous limitations surrounding the time required to generate autologous EBV-CTLs (2–3 months) in critically ill patients at risk for rapid progression of disease have now been addressed with availability of third party, HLA compatible EBV-CTLs with favorable safety profile and outcomes (62, 65). While a clear benefit to adoptive cell therapy is the potential to establish viral specific T-cell memory while avoiding immune-ablation and organ toxicity seen in chemotherapy (66), patients who receive EBV-CTLs may continue to require long-term immunosuppression to prevent allograft rejection, which poses an increased risk for invasive infections and additional malignancies. More research in larger populations is necessary to understand risks, benefits and long-term effects of EBV-CTLs in pediatric SOT patients (62).

## Prognosis and long-term outcomes

Published data in all pediatric transplant recipients suggest a multitude of parameters associated with poor prognosis, including: advanced disease, multifocal and extra-nodal disease, CNS involvement, allograft involvement and high EBV load at the time of diagnosis. Morphologically, CD20-negative, EBV-negative, monomorphic subtype and late-onset PTLD are all associated with less favorable outcomes (67); as well as poor response to initial therapy (68). Pediatric kidney transplant recipients may have a more favorable outcome than their lung, liver, or HSCT counterparts; and overall, children diagnosed with PTLD have a better prognosis than adults (10, 68–70). Survival data in childhood PTLD is limited to small case series and studies across all allograft types and varied clinical and histological presentations; but in general, 5-year survival rates are estimated at 53%–80% (6, 59, 67, 68, 71–73). More long-term follow-up studies are necessary to determine late-onset morbidity and mortality associated with PTLD, including identifying parameters for safe re-transplantation (74, 75).

There are currently two major gaps in research of PTLD. First, not all EBV is the same. Many patients are chronic EBV carriers and never develop PTLD. A better understanding of the viral and host factors and immune responses that determine whether EBV-positive PLTD develops is critically needed (76). In addition, PTLD genotypes and phenotypes are vast, and once PTLD develops, there are no tailored protocols. Further research to improve characterization of the tumor microenvironment would help advance tailored therapeutic strategies to optimize outcomes and reduce adverse effects, ideally minimizing risk to the allograft (77).

Overall, PTLD is a rare but serious complication after kidney transplantation, and many questions remain unanswered given the wide spectrum of disease presentation with variable phenotypes, organ involvement and the complex interplay among factors that determine treatment and prognosis. Until vaccines are proven safe and effective and become readily available, attention must continue to be focused on prevention and management.
